# Supplementary material for: Frailty Prevalence and Association with Clinical Outcomes in Interstitial Lung Disease, Asthma, and Pleural Disease
Source: Geriatrics (Basel). 2023 Aug 13;8(4):82. doi: 10.3390/geriatrics8040082 (PMC10454934; doi:10.3390/geriatrics8040082)
Supplement: Supplementary file 1 [file geriatrics-08-00082-s001.zip › geriatrics-2539958-supplementary.pdf]

SUPPLEMENTARY MATERIAL (File S1)

S1 Figure 1A. PRISMA Flowchart of the included studies on Asthma.

S1 Figure 1B. PRISMA Flowchart of the included studies on Pleural Disease.

S1 Table 1A. Newcastle-Ottawa Score for the included studies on ILD.

S1 Table 1B. Newcastle-Ottawa Score (NOS) domain description.

S1 Table 1C. Frailty instruments used in the Included Studies on ILD and Asthma.

S1 Table 2A. Newcastle-Ottawa Score for the included studies on Asthma.

S1 Figure 3. Forest plot describing effect of frailty, pre-frailty, and non-frailty on all-cause mortality in ILD.

S1 Table 3. Search strategy.

S1 Table 4. PRISMA 2020 Checklist.

S1 Figure 1A. PRISMA Flowchart of the included studies on Asthma.

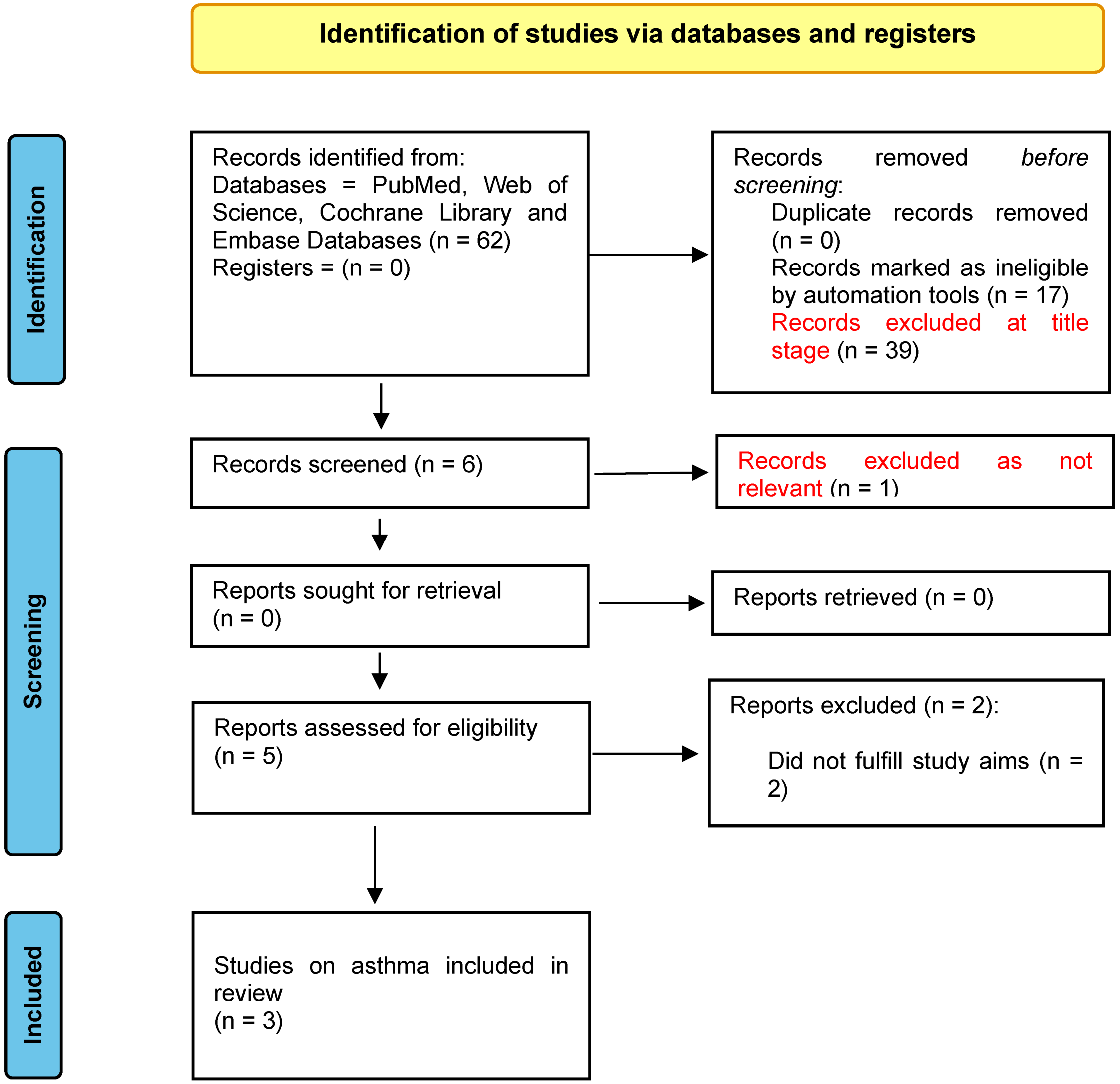

From: Page MJ, McKenzie JE, Bossuyt PM, Boutron I, Hoffmann TC, Mulrow CD, *et al*. The PRISMA 2020 statement: an updated guideline for reporting systematic reviews. BMJ 2021;372:n71.

S1 Figure 1B. PRISMA Flowchart of the included studies on Pleural Disease.

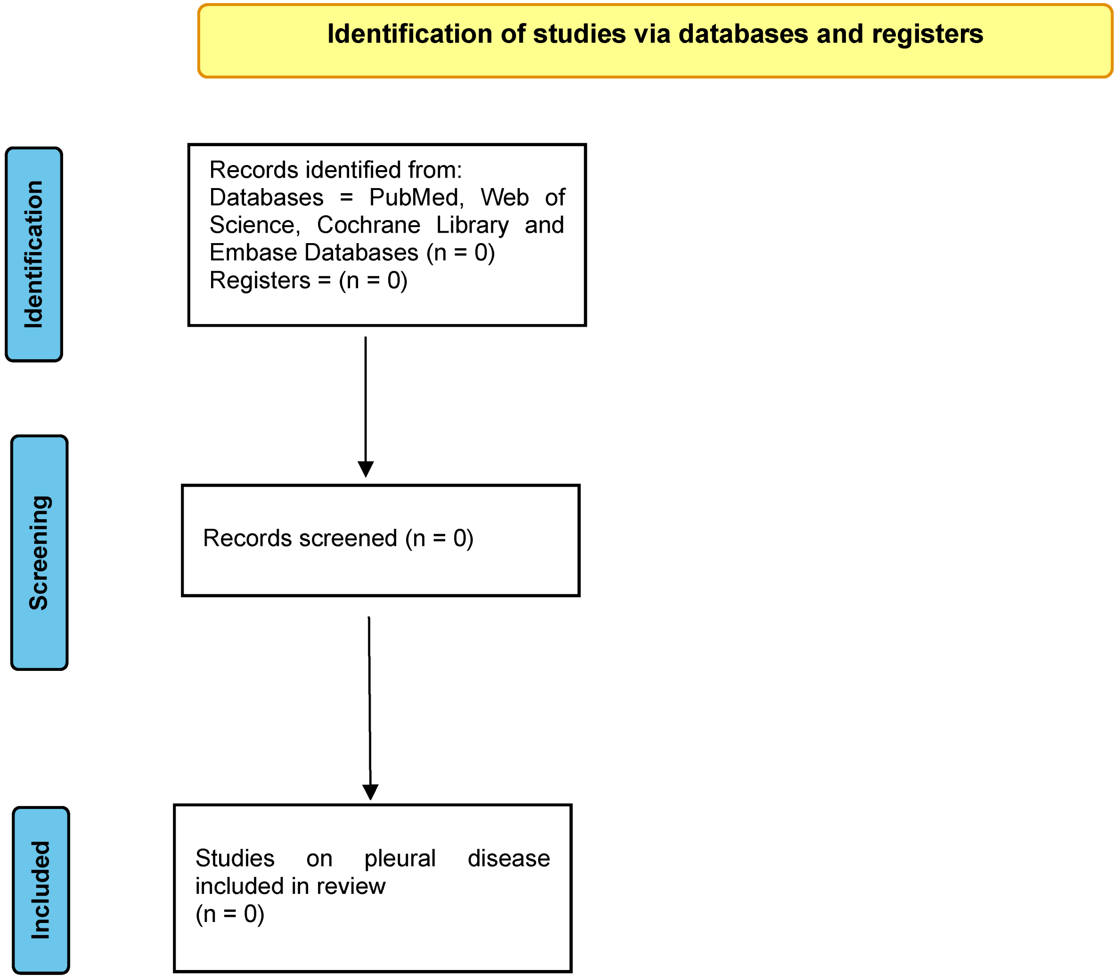

From: Page MJ, McKenzie JE, Bossuyt PM, Boutron I, Hoffmann TC, Mulrow CD, *et al*. The PRISMA 2020 statement: an updated guideline for reporting systematic reviews. BMJ 2021;372:n71.

S1 Table 1A. Newcastle-Ottawa Score for the included studies on ILD.

	Selection &				Comparability *		Outcome *			Quality Assessment **	Notes
	**1**	**2**	**3**	**4**	**1**	**2**	**1**	**2**	**3**		
Farooqi MAM 2021	1	1	1	1	1	1	1	1	1	Good	1.7-year follow up
Guler SA 2020	1	1	1	1	1	1	1	1	1	Good	17 months follow up
Sheth JS 2019	1	1	1	1	1	0	1	0	0	Fair	No follow up data
Guler SA 2017	1	1	1	1	1	0	1	0	0	Fair	No follow up data
Milne KM 2017	1	1	1	1	1	0	1	0	0	Fair	No follow up data
Labreque F-P 2022	1	1	1	1	1	1	1	0	0	Good	No follow up data

Domain scored&: Good (3+); fair (2); poor (0-1).
Domain scored*: Good (2-3); fair (1); poor (0).
**For a study to be classed as good quality, it had to score 'good' for every domain, two domains were deemed as fair quality and one or no domains as poor quality

S1 Table 1B. Newcastle-Ottawa Score (NOS) domain description.

Domain		Domain description
Selection	1	Representativeness of the exposed cohort of the overall community
	2	Selection of the non exposed cohort from the same community as the exposed cohort
	3	Ascertainment of exposure; reliable method of data collection used
	4	Demonstration that outcome of interest was not present at start of study
Comparability	1	Comparability of groups
	2	Adjusted in the analysis
Outcome	1	Assessment of outcome; reliable method of data collection used
	2	Was follow-up long enough for outcomes to occur?
	3	Adequacy of follow up of cohorts; were all subjects accounted for?

S1 Table 1C. Frailty instruments used in the Included Studies on ILD and Asthma.

References/Year	Frailty Scale	Items measured	Scoring	Administration
Fried et al. 2001	**Fried Frailty phenotype**<br><br>(Physical Frailty Phenotype)	5 domains:<br><br>Slowness<br>Physical activity<br>Weight loss<br>Exhaustion<br>Weakness	Score range: 0 to 5.<br><br>Frail = ≥3 criteria<br><br>Pre-frail = 1-2 criteria<br><br>Non-frail = 0	Physician and self-reported
Mitnitski et al. 2001; Rockwood et al. 2007	**Frailty Index**<br><br>(Deficit Accumulation Index)	Scales vary in content and number of items, generally 30-70.<br><br>Multiple domains including laboratory findings, physical function disabilities, diseases, symptoms, sensory difficulties, cognition difficulties	Number of deficits present and divided by the number of deficits considered. Higher proportion = higher level of frailty.	Physician
Tomata et al. 2011	**Kihon Checklist**	25-item questionnaire including 7 domains: instrumental activity of daily living, social activity of daily living, physical strength, nutritional status, oral function, cognitive status, depression risk	Non-frail = 0-3<br><br>Pre-frail = 4-7<br><br>Frail = ≥ 8	Physician

S1 Table 2A. Newcastle-Ottawa Score for the included studies on Asthma.

	Selection &				Comparability *		Outcome *			Quality Assessment **	Notes
	**1**	**2**	**3**	**4**	**1**	**2**	**1**	**2**	**3**		
Landre’ B 2020	0	1	1	1	0	0	1	0	0	Fair	Self-reported diagnosis of asthma, 26-year follow up data, prevalence of frailty in subjects with or without current asthma
Kusunose M 2021	1	1	1	1	1	0	1	0	0	Fair	Diagnosis of asthma according to guidelines; no follow up data
Hanlon P 2018	0	1	1	1	1	1	1	1	1	Good	Self-reported diagnosis of asthma, 7-year follow up, prevalence of frailty in subjects with asthma, all-cause mortality data in frail group not adjusted for diagnosis of asthma

Domain scored &: Good (3+); fair (2); poor (0-1)
Domain scored *: Good (2-3); fair (1); poor (0)
**For a study to be classed as good quality, it had to score ‘good’ for every domain, two domains were deemed as fair quality and one or no domains as poor quality

S1 Figure 3. Forest plot describing effect of frailty, pre-frailty, and non-frailty on all-cause mortality in ILD.

Study or Subgroup	Frail Events	Frail Total	Non-frail/pre-frail Events	Non-frail/pre-frail Total	Weight	Odds Ratio M-H, Random, 95% CI
Farooqi 2021	17	123	39	340	49.2%	1.24 [0.67, 2.28]
Guler 2020	62	272	19	268	50.8%	3.87 [2.24, 6.68]
**Total (95% CI)**		**395**		**608**	**100.0%**	**2.21 [0.72, 6.76]**
Total events	79		58			

Heterogeneity: Tau² = 0.57; Chi² = 7.47, df = 1 (P = 0.006); I² = 87%
Test for overall effect: Z = 1.39 (P = 0.17)

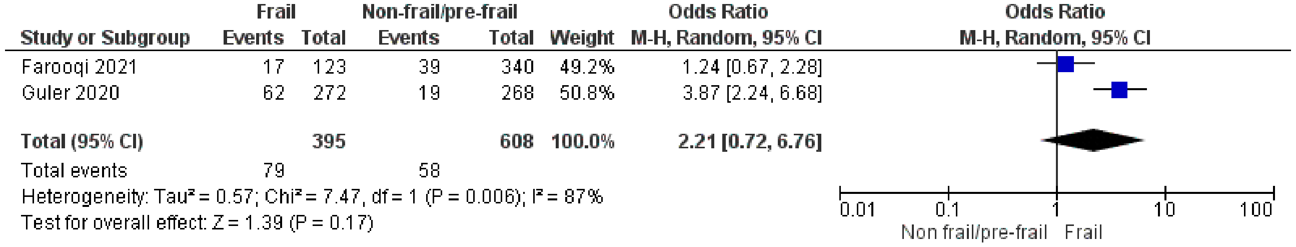

Panel A. Frailty *vs* Non-Frailty/Pre-Frailty and Long-term Mortality in ILD patients.

Study or Subgroup	Frail/pre-frail Events	Frail/pre-frail Total	Non frail Events	Non frail Total	Weight	Odds Ratio M-H, Random, 95% CI
Farooqi 2021	53	381	3	82	40.3%	4.26 [1.30, 13.97]
Guler 2020	62	391	19	149	59.7%	1.29 [0.74, 2.24]
**Total (95% CI)**		**772**		**231**	**100.0%**	**2.09 [0.65, 6.73]**
Total events	115		22			

Heterogeneity: Tau² = 0.52; Chi² = 3.32, df = 1 (P = 0.07); I² = 70%
Test for overall effect: Z = 1.23 (P = 0.22)

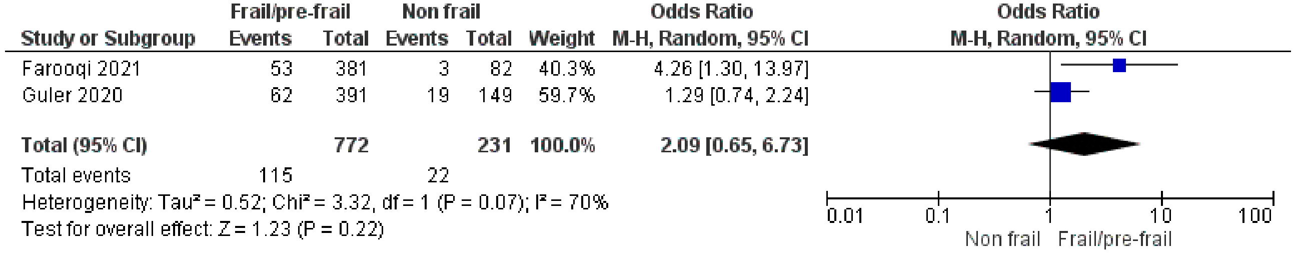

Panel B. Frailty/Pre-Frailty *vs* Non-Frailty and Long-term Mortality in ILD patients

S1 Table 3. Search strategy for ILD.

Embase Classic+Embase <1947 to 2022 October 25>

Search strategy:

----

1 (frailty and interstitial lung disease).mp. [mp=title, abstract, heading word, drug trade name, original title, device manufacturer, drug manufacturer, device trade name, keyword heading word, floating subheading word, candidate term word] 77

2 (frailty and idiopathic pulmonary fibrosis).mp. [mp=title, abstract, heading word, drug trade name, original title, device manufacturer, drug manufacturer, device trade name, keyword heading word, floating subheading word, candidate term word] 52

3 (frailty and non-specific interstitial pneumonia).mp. [mp=title, abstract, heading word, drug trade name, original title, device manufacturer, drug manufacturer, device trade name, keyword heading word, floating subheading word, candidate term word] 0

4 (frailty and Chronic Hypersensitivity Pneumonitis).mp. [mp=title, abstract, heading word, drug trade name, original title, device manufacturer, drug manufacturer, device trade name, keyword heading word, floating subheading word, candidate term word] 2

5 (frailty and Systemic sclerosis-associated interstitial lung disease).mp. [mp=title, abstract, heading word, drug trade name, original title, device manufacturer, drug manufacturer, device trade name, keyword heading word, floating subheading word, candidate term word] 3

6 (frailty and Connective tissue disease-associated interstitial lung disease).mp. [mp=title, abstract, heading word, drug trade name, original title, device manufacturer, drug manufacturer, device trade name, keyword heading word, floating subheading word, candidate term word] 1

S1 Table 4.

PRISMA 2020 Checklist

Section and Topic	Item #	Checklist item	Location where item is reported
**TITLE**			
Title	1	Identify the report as a systematic review.	
**ABSTRACT**			
Abstract	2	See the PRISMA 2020 for Abstracts checklist.	
**INTRODUCTION**			
Rationale	3	Describe the rationale for the review in the context of existing knowledge.	
Objectives	4	Provide an explicit statement of the objective(s) or question(s) the review addresses.	
**METHODS**			
Eligibility criteria	5	Specify the inclusion and exclusion criteria for the review and how studies were grouped for the syntheses.	
Information sources	6	Specify all databases, registers, websites, organisations, reference lists and other sources searched or consulted to identify studies. Specify the date when each source was last searched or consulted.	
Search strategy	7	Present the full search strategies for all databases, registers and websites, including any filters and limits used.	
Selection process	8	Specify the methods used to decide whether a study met the inclusion criteria of the review, including how many reviewers screened each record and each report retrieved, whether they worked independently, and if applicable, details of automation tools used in the process.	
Data collection process	9	Specify the methods used to collect data from reports, including how many reviewers collected data from each report, whether they worked independently, any processes for obtaining or confirming data from study investigators, and if applicable, details of automation tools used in the process.	
Data items	10a	List and define all outcomes for which data were sought. Specify whether all results that were compatible with each outcome domain in each study were sought (e.g. for all measures, time points, analyses), and if not, the methods used to decide which results to collect.	
	10b	List and define all other variables for which data were sought (e.g. participant and intervention characteristics, funding sources). Describe any assumptions made about any missing or unclear information.	
Study risk of bias assessment	11	Specify the methods used to assess risk of bias in the included studies, including details of the tool(s) used, how many reviewers assessed each study and whether they worked independently, and if applicable, details of automation tools used in the process.	
Effect measures	12	Specify for each outcome the effect measure(s) (e.g. risk ratio, mean difference) used in the synthesis or presentation of results.	
Synthesis methods	13a	Describe the processes used to decide which studies were eligible for each synthesis (e.g. tabulating the study intervention characteristics and comparing against the planned groups for each synthesis (item #5)).	

Section and Topic	Item #	Checklist item	Location where item is reported
	13b	Describe any methods required to prepare the data for presentation or synthesis, such as handling of missing summary statistics, or data conversions.	
	13c	Describe any methods used to tabulate or visually display results of individual studies and syntheses.	
	13d	Describe any methods used to synthesize results and provide a rationale for the choice(s). If meta-analysis was performed, describe the model(s), method(s) to identify the presence and extent of statistical heterogeneity, and software package(s) used.	
	13e	Describe any methods used to explore possible causes of heterogeneity among study results (e.g. subgroup analysis, meta-regression).	
	13f	Describe any sensitivity analyses conducted to assess robustness of the synthesized results.	
Reporting bias assessment	14	Describe any methods used to assess risk of bias due to missing results in a synthesis (arising from reporting biases).	
Certainty assessment	15	Describe any methods used to assess certainty (or confidence) in the body of evidence for an outcome.	
**RESULTS**			
Study selection	16a	Describe the results of the search and selection process, from the number of records identified in the search to the number of studies included in the review, ideally using a flow diagram.	
	16b	Cite studies that might appear to meet the inclusion criteria, but which were excluded, and explain why they were excluded.	
Study characteristics	17	Cite each included study and present its characteristics.	
Risk of bias in studies	18	Present assessments of risk of bias for each included study.	
Results of individual studies	19	For all outcomes, present, for each study: (a) summary statistics for each group (where appropriate) and (b) an effect estimate and its precision (e.g. confidence/credible interval), ideally using structured tables or plots.	
Results of syntheses	20a	For each synthesis, briefly summarise the characteristics and risk of bias among contributing studies.	
	20b	Present results of all statistical syntheses conducted. If meta-analysis was done, present for each the summary estimate and its precision (e.g. confidence/credible interval) and measures of statistical heterogeneity. If comparing groups, describe the direction of the effect.	
	20c	Present results of all investigations of possible causes of heterogeneity among study results.	
	20d	Present results of all sensitivity analyses conducted to assess the robustness of the synthesized results.	
Reporting biases	21	Present assessments of risk of bias due to missing results (arising from reporting biases) for each synthesis assessed.	
Certainty of evidence	22	Present assessments of certainty (or confidence) in the body of evidence for each outcome assessed.	
**DISCUSSION**			
Discussion	23a	Provide a general interpretation of the results in the context of other evidence.	

Section and Topic	Item #	Checklist item	Location where item is reported
	23b	Discuss any limitations of the evidence included in the review.	
	23c	Discuss any limitations of the review processes used.	
	23d	Discuss implications of the results for practice, policy, and future research.	
**OTHER INFORMATION**			
Registration and protocol	24a	Provide registration information for the review, including register name and registration number, or state that the review was not registered.	
	24b	Indicate where the review protocol can be accessed, or state that a protocol was not prepared.	
	24c	Describe and explain any amendments to information provided at registration or in the protocol.	
Support	25	Describe sources of financial or non-financial support for the review, and the role of the funders or sponsors in the review.	
Competing interests	26	Declare any competing interests of review authors.	
Availability of data, code and other materials	27	Report which of the following are publicly available and where they can be found: template data collection forms; data extracted from included studies; data used for all analyses; analytic code; any other materials used in the review.	

From: Page MJ, McKenzie JE, Bossuyt PM, Boutron I, Hoffmann TC, Mulrow CD, et al. The PRISMA 2020 statement: an updated guideline for reporting systematic reviews. BMJ 2021;372:n71. doi: 10.1136/bmj.n71

For more information, visit: http://www.prisma-statement.org/
